# Estimation of Viral Aerosol Emissions From Simulated Individuals With Asymptomatic to Moderate Coronavirus Disease 2019

**DOI:** 10.1001/jamanetworkopen.2020.13807

**Published:** 2020-07-27

**Authors:** Michael Riediker, Dai-Hua Tsai

**Affiliations:** 1 Swiss Centre for Occupational and Environmental Health, Winterthur, Switzerland; 2 Department of Child and Adolescent Psychiatry and Psychotherapy, University Hospital of Psychiatry, University of Zurich, Zurich, Switzerland

## Abstract

**Importance:**

Individuals with asymptomatic or mild coronavirus disease 2019 (COVID-19) have been reported to frequently transmit the disease even without direct contact. The severe acute respiratory syndrome coronavirus 2 has been found at very high concentrations in swab and sputum samples from such individuals.

**Objective:**

To estimate the virus levels released from individuals with asymptomatic to moderate COVID-19 into different aerosol sizes by normal breathing and coughing, and to determine what exposure could result from this in a room shared with such individuals.

**Design, Setting, and Participants:**

This mathematical modeling study combined the size-distribution of exhaled breath microdroplets for coughing and normal breathing with viral swab and sputum concentrations as approximation for lung lining liquid to obtain an estimate of emitted virus levels. Viral data were obtained from studies published as of May 20, 2020. The resulting emission data fed a single-compartment model of airborne concentrations in a room of 50 m^3^, the size of a small office or medical examination room.

**Main Outcomes and Measures:**

Modeling was used to estimate the viral load emitted by individuals breathing normally or coughing, and the concentrations expected in the simulated room at different ventilation rates.

**Results:**

The mean estimated viral load in microdroplets emitted by simulated individuals while breathing regularly was 0.0000049 copies/cm^3^, with a range of 0.0000000049 to 0.637 copies/cm^3^. The corresponding estimates for simulated coughing individuals were a mean of 0.277 copies/cm^3^ per cough, with a range of 0.000277 to 36 030 copies/cm^3^ per cough. The estimated concentrations in a room with an individual who was coughing frequently were very high, with a maximum of 7.44 million copies/m^3^ from an individual who was a high emitter. However, regular breathing from an individual who was a high emitter was modeled to result in lower room concentrations of up to 1248 copies/m^3^.

**Conclusions and Relevance:**

In this modeling study, breathing and coughing were estimated to release large numbers of viruses, ranging from thousands to millions of virus copies per cubic meter in a room with an individual with COVID-19 with a high viral load, depending on ventilation and microdroplet formation process. The estimated infectious risk posed by a person with typical viral load who breathes normally was low. The results suggest that only few people with very high viral load pose an infection risk in poorly ventilated closed environments. These findings suggest that strict respiratory protection may be needed when there is a chance to be in the same small room with an individual, whether symptomatic or not, especially for a prolonged period.

## Key Points

**Question:**

What is the estimated viral load released from an individual with coronavirus disease 2019 (COVID-19) by breathing and coughing, and what is the resulting concentration in a room?

**Findings:**

In this mathematical modeling study, breathing and coughing by a simulated individual with COVID-19 were estimated to release large numbers of viruses in a poorly ventilated room with a coughing person. However, the estimated infectious risk posed by a person with typical viral load who breathes normally was low, and only few people with very high viral load posed an infection risk in a poorly ventilated closed environment.

**Meaning:**

These results may partially explain the observed rates of transmission and suggest that there is a need for strict respiratory protection when people are in the same room with an individual with COVID-19.

## Teaser

This mathematical modeling study estimates viral aerosol emissions from simulated individuals with asymptomatic to moderate coronavirus disease 2019 (COVID-19).

## Introduction

The novel coronavirus disease 2019 (COVID-19) emerged in late 2019 in Wuhan, China,^1^ and eventually spread to the rest of the world. It is caused by a novel type of coronavirus, the severe acute respiratory syndrome coronavirus 2 (SARS-CoV-2).^2^ The host-receptor for SARS-CoV-2 was found to be angiotensin converting enzyme 2, which is present in cells of the lungs and airways.^3^ In the early phase of the outbreak, a large number of patients who were hospitalized for other reasons^4^ and a considerable proportion of medical staff treating them^5^ contracted COVID-19. However, the infection rate among medical staff corresponded to community rates when respiratory personal protective equipment was used at work.^6,7^ Additionally, a series of community-transmissions were reported that were associated with individuals who had no apparent symptoms.^8,9,10,11^ The estimated infection rates are currently in the range of 1% in the community and 10% within households.^12,13,14,15^ However, during super-spreading events in situations where many people engaged in loud voice activities gathered in closed rooms for prolonged time, such as a restaurant,^16^ a call center,^17^ a dermatologists’ scientific board meeting,^18^ and a choir rehearsal,^19^ infection rates higher than 75% have been reported. Notably, the individuals involved in the choir rehearsal event tried to follow social distancing and hand washing guidelines.^19^ These super-spreading events suggest that the airborne route may represent a virus transmission form in some indoor situations. Indeed, a study conducted in a Wuhan hospital^20^ found low airborne concentrations of the virus in the intensive care unit and in medical staff rooms. Several studies have reported very high concentrations of SARS-CoV-2 in samples taken in the nose, throat, and saliva from individuals with mild or asymptomatic COVID-19,^11,21,22,23^ and high viral loads during antiviral treatment.^24^ All of these reports have raised questions regarding whether viral transmission could occur via the air.

When coughing, humans release thousands of microdroplets per cubic centimeter in the size range of 0.6 to 15 μm, with the droplet concentration increasing with cough flow rate.^25^ However, regular breathing also leads to some microdroplet production, which is attributed to fluid film rupture in the respiratory bronchioles during inhalation leading to the formation of droplets that are released during exhalation.^26^ The size of these droplets is mostly smaller than 1 μm.^27^ The mode of droplet generation implies that they consist of lung lining liquid, including dispersed viruses. Indeed, human volunteers exposed to virus-sized nanoparticles show nanoscaled particles in their exhaled breath.^28,29^ Also, the described size distribution of particles emitted from coughing as well as regular respiration suggests that an important proportion of particles will remain airborne for many hours in turbulent conditions.^30^

This study aimed to estimate the cumulative viral load released from simulated individuals with asymptomatic to moderate COVID-19 in different microdroplet sizes via respiration and coughing. We used this information to make a risk appraisal for the simulated low, typical, or high emitter that is either breathing normally or coughing in a room operated at different air exchange rates. We chose a room size that is similar to a medical examination room or an office shared by 2 to 3 people.

## Methods

### Concept

The release of viruses from simulated individuals was modeled by first calculating the viral load per exhaled microdroplets formed during normal breathing and while coughing. The resulting size distribution provided an initial estimate of the concentration of SARS-CoV-2 virus copies released by a regularly breathing or coughing simulated individual. This viral emission factor was then fed into a well-mixed 1-compartment model to simulate the situation in a closed room with different ventilation air exchange rates. This study follows the concept of Strengthening the Reporting of Empirical Simulation Studies (STRESS) guideline.^31^ This mathematical modeling corresponds to a meta-analysis. We used aggregate data from previous clinical studies in which informed consent had already been obtained by the trial investigators; therefore, our study was exempt from ethics approval, according to the Common Rule.

### Data Sources

Data on the number of viral copies present in sputum and swab samples of individuals with COVID-19 were used to estimate the SARS-CoV-2 viral load present in the lining liquid of respiratory bronchioles, based on available studies published as of May 20, 2020.^11,21,22,23,24,32^ Specifically, we used 1000 copies/mL to represent an individual who was a low emitter, 10^6^ copies/mL to represent a typical emitter, and 1.3 × 10^11^ copies/mL to represent a high emitter.

Exhaled microdroplet size distributions and numbers were retrieved from published studies on healthy persons coughing^25^ and breathing normally.^26^ Both studies assessed the size, number, and distribution of freshly emitted microdroplets. The concentration of viral copies in each microdroplet size was calculated from the volume of the microdroplets, the actual count number in each size, and the aforementioned virus-load per milliliter of sputum. The viral load in the actual microdroplet counts in each microdroplet size was then used to calculate the total viral concentration. The cumulative emissions in the particulate matter 10 μm or less in diameter (PM_10_) fraction were summed after applying the standard size fractionation curves^33^ to the microdroplet distribution.

### Model

A 1-compartment model^34^ estimated the virus load concentration *C* for a perfectly mixed room of volume *V_R_* of 50 m^3^ with one simulated individual as source, using the following mass-balance:
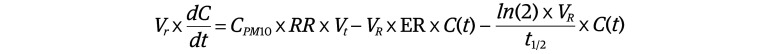
The emission rate was calculated from the concentration *C_PM10_*, the viral load in the PM_10_-size range, which are particles collected with a 50% efficiency cutoff at 10 μm aerodynamic diameter; and a respiratory rate (RR) of 15 breaths per minute at a tidal volume of *V_t_* of 500 mL per breath. Air exchange rates used were 1-, 3-, 10-, and 20-times per hour. The virus’ half-life (*t_½_*) of 1.1 hours was obtained from an experimental study about the persistence of SARS-CoV-2 on surfaces and when airborne,^35^ tested by assessing the 50% tissue culture infective dose (*TCID_50_*). The model for coughing was identical, except that coughing was assumed to happen every 30 seconds at a volume of 250 mL, as described for a person with a chronic dry cough (although not with COVID-19).^36^

### Statistical Analysis

All statistics and models were calculated using Stata/SE version 15.1 (StataCorp). Robust data reported include estimated means and ranges. The models and code are available on request. Data were analyzed from March 30 to May 27, 2020.

## Results

### Emissions From Simulated Individuals Breathing Normally

**Figure 1.**
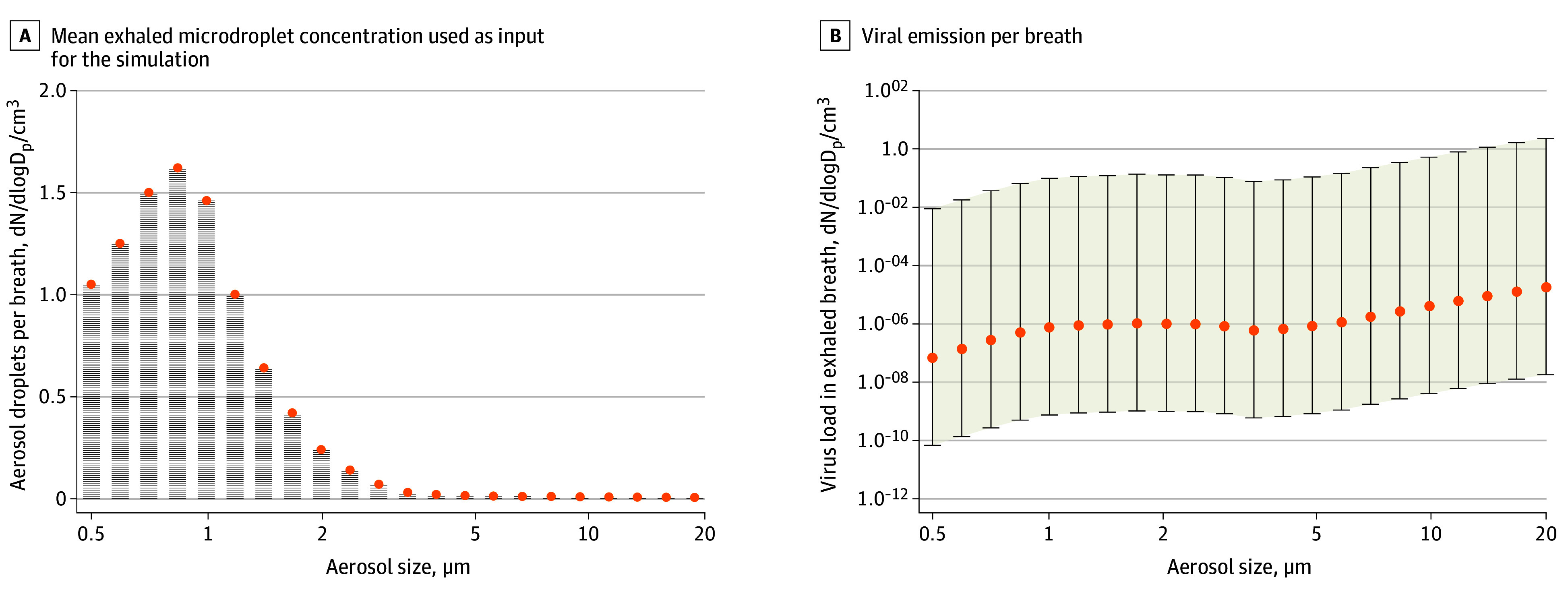
Size Distribution of Exhaled Microdroplets and Resulting Viral Emissions During Regular Breathing A, The typical exhaled microdroplet concentration used as input for the simulation. B, The modeled viral emission per breath for typical (orange), high, and low (whiskers) emitters. dN/dlogD_p_/cm^3^ is the number concentration normalized by the aerosol size-bin width.

To estimate the virus emissions from simulated individuals breathing normally, we first calculated the viral load for the microdroplet size distribution. Figure 1 shows that the highest virus load is present in the largest microdroplet size. The cumulative total emission per breath was 0.0000000049 copies/cm^3^ of air for a low emitter, 0.0000049 copies/cm^3^ for a typical emitter, and 0.637 copies/cm^3^ for a high emitter. The cumulative emissions in the PM_10_ fraction were approximately one-third of these values, with 0.0000017 copies/cm^3^ per breath for a typical emitter and 0.226 copies/cm^3^ per breath for a high emitter.

### Emission From a Coughing Simulated Individual

**Figure 2.**
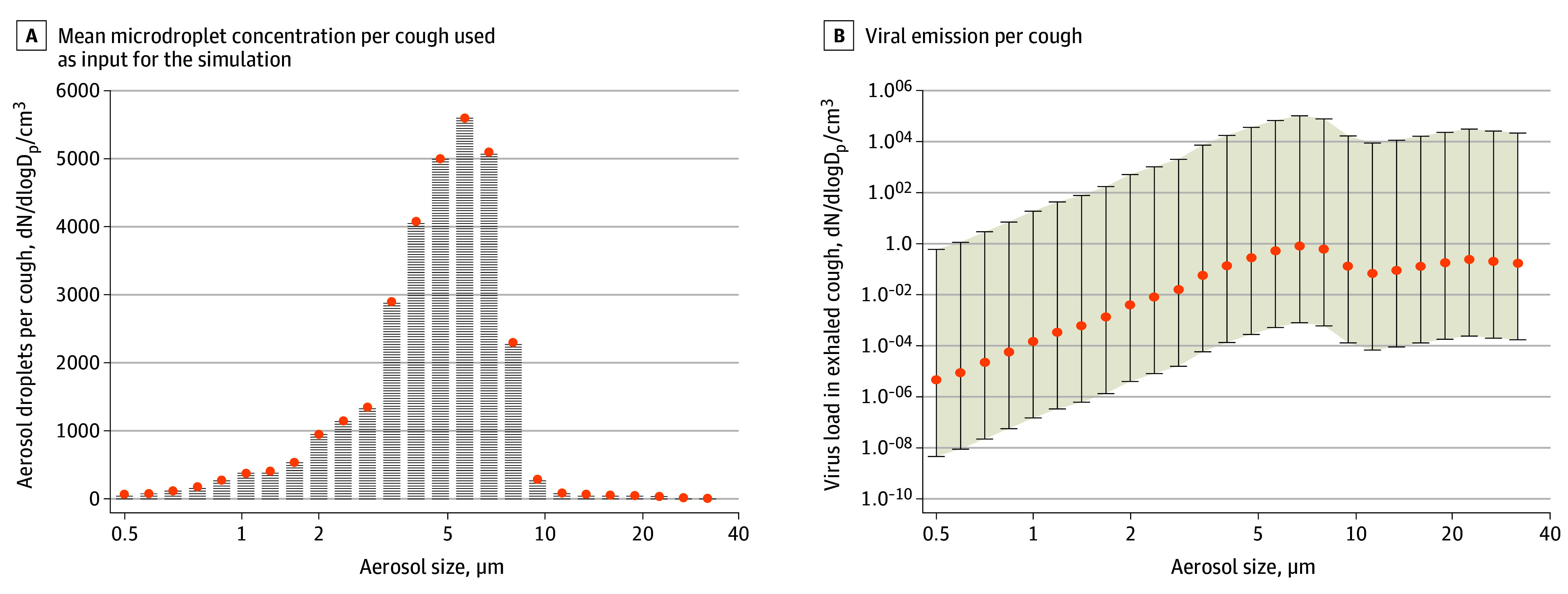
Size Distribution of Exhaled Microdroplets and Resulting Viral Emissions During Coughing A, The typical exhaled microdroplet concentration used as input for the simulation. B, The modeled viral emission per breath for typical (orange), high, and low (whiskers) emitters. dN/dlogD_p_/cm^3^ is the number concentration normalized by the aerosol size-bin width.

We then estimated the virus emissions from a simulated coughing individual (Figure 2). The cumulative total emission per cough was 0.000277 copies/cm^3^ for a low emitter, 0.277 copies/cm^3^ for a typical emitter, and 36 030copies/cm^3^ for a high emitter. The cumulative emissions in the PM_10_ fraction were approximately half of these values, with 0.156 copies/cm^3^ per cough for a typical emitter and 20 221 copies/cm^3^ per cough for a high emitter.

### Exposure Estimation for Bystanders

**Figure 3.**
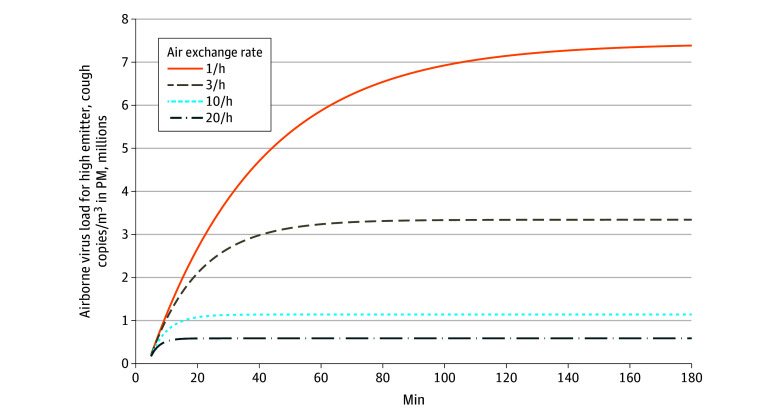
Temporal Course of Airborne Virus Load in a Perfectly Mixed Room of 50 m^3^ The simulation estimated the concentration in a closed room for different air exchange rates. The emitter was assumed to have a high virus load in the lungs and to be coughing intermittently every 30 seconds.

To estimate the exposure for bystanders spending time in the same room as an individual with COVID-19, we calculated the time-course of the viral load in the thoracic size fraction for small droplets released from a high emitter either breathing normally or coughing. Figure 3 shows the results for a high-emitting simulated individual coughing frequently.

For a typical hospital ventilation situation of 10 air exchanges per hour, the concentration plateaus after approximately 30 minutes, while for a typical office with 3 air exchanges per hour, concentrations continue to increase for more than 1 hour. In this model, concentrations scale linearly with the simulated individual emission rate; the plateau concentrations for different emitting simulated individuals and ventilation types are summarized in the Table.

**Table.** Plateau Concentration for Different Combinations of Air Exchange Rate, Emission Form, and Emitter Type

Measure	Air exchange rate, times/h			
	1	3	10	20
Time until 99% of plateau, min	169	77	26	14
Airborne viral concentration at plateau, copies/m^3^				
Regular breathing				
Low emitter	0.000009598	0.000004310	0.000001472	0.000000758
Typical emitter	0.009598	0.004310	0.001472	0.000758
High emitter	1247.7	560.3	191.3	98.6
Frequent coughing^a^				
Low emitter	0.057251	0.025709	0.008779	0.004524
Typical emitter	57.251	25.709	8.779	4.524
High emitter	7 442 598	3 342 148	1 141 326	588 093

^a^ Defined as coughing every 30 seconds.

## Discussion

In this modeling study, breathing and coughing were estimated to release large numbers of viruses, ranging from thousands to millions of virus copies per cubic meter in a room with an individual with COVID-19 with a high viral load, depending on ventilation and microdroplet formation process. The estimated infectious risk posed by a person with typical viral load who breathes normally was low. The results suggest that only a few people with very high viral load posed an infection risk in the poorly ventilated closed environment simulated in this study.

An individual with COVID-19 with a high viral load would be expected to release a high number of viruses in the form of airborne microdroplets, especially when the individual is coughing. While the bigger portion of the emitted viral load is in the form of large droplets that can deposit rapidly, there is also an important portion in the smaller size fractions. Small microdroplets can remain airborne for an extended time^30^ and are very effective at reaching the lungs.^37^

One study assessed airborne SARS-CoV-2 levels in a hospital in Wuhan, China, and found concentrations in the range of 20 copies/m^3^ in medical staff offices and meeting rooms.^20^ This concentration agrees with what our modeling would suggest for a small room with a regularly breathing person who was asymptomatic having a viral load above a typical emitter.

A typical person breathes approximately 0.5 m^3^ per hour in resting state,^38^ which can rapidly increase to several cubic meters per hour during exercise.^39^ Thus, a person spending time in a room with an individual emitting at a typical rate and breathing normally has the chance of inhaling only a few copies of the virus when keeping distance from that person. However, the risk may be higher in the presence of an individual with a high emitting rate and if the individual is coughing. A review of a wide range of respiratory viruses suggests that the infective dose is often low. Sometimes as few as a few hundred units of active virus^40^ seem sufficient to cause disease. Thus, our modeling suggests that there is a risk of infection for a person spending an extended period in a small room with an individual with COVID-19 who has an elevated viral load, even if the distance is too large for direct transmission. The risk may be higher if the individual is coughing.

People who are high emitters are not very common in the population. However, if such a person is engaged in activities, such as loud speaking or singing, microdroplet formation and thus viral emissions can increase by 1 to 2 orders of magnitude.^41^ This may help explain the occasional super-spreading events in crowded situations involving loud voices.^16,17,18,19^

The occasionally very high virus load in exhaled respiratory microdroplets proposed by our assessment may be an explanation of why COVID-19 was associated with more transmissions to hospital staff than the rate expected from SARS.^4^ While wearing a surgical face mask can be an effective source control,^42^ the protective factors may still be insufficient if an extended amount of time is spent in the same room with a coughing individual who has a high viral load, especially if the room is small and the ventilation low. Increasing ventilation can help to some extent, but it is not sufficient in a room the size of a typical office or medical examination room. Note also that ventilation design for hospitals is complex and not always functioning as intended.^43^

The implications of these findings for everyday life and the workplace are that individuals may be at risk of infection if they spend more than a few minutes in a small room with a person who is infected with COVID-19 and has a high viral load. Sharing a workplace in a small room with a person with asymptomatic COVID-19 is not advised. This implies that workplaces should not be shared as long as there are no rapid tests to differentiate between individuals without COVID-19 and individuals with asymptomatic COVID-19. Medical staff are advised to wear the best possible respiratory protection whenever in the same room as an individual, especially when the individual is coughing, in which case eye protection is advised as well.^44^ In addition, every individual, even those who are asymptomatic, should wear a well-fitting surgical face mask to reduce emissions, which would increase the overall protection for the medical staff.^42^

### Limitations

Our assessment has a number of limitations. First, the estimated virus levels depend on the number of virus copies produced by an individual with COVID-19. We used sputum and swab data from a few well-described peer-reviewed studies,^11,21,22,23,24,32^ assuming that they provide reasonable approximations for the virus load in the respiratory bronchioles, the space where most respiratory microdroplets are formed. Our high emitter estimates would be 100-fold higher if the most extreme viral data were combined with microdroplet super emissions.^22,41^ Second, we used information about virus copies but compared the results with TCID_50_. Research on other virus types suggests that the number of virus copies and TCID_50_ are comparable.^45^ However, it would be important to confirm this for SARS-CoV-2. Third, for breath and cough microdroplets release, we used data collected in experimental setups involving healthy young participants. However, microdroplet formation is influenced by surface tension of the lung lining liquid.^46^ It is likely that microdroplet formation would be altered in individuals with COVID-19, but it is not clear in which direction. Fourth, microdroplets will shrink in dry air,^47^ resulting in a shift to smaller particle sizes. This will not directly change the number of copies in the PM_10_ range but simply increase the concentration of the viral load per microdroplet. While we addressed passivation of viruses in the air by using the documented half-life,^35^ it is still possible that viruses in smaller droplets are more quickly passivated because of shorter diffusion distances for airborne oxidants and faster increasing salinity. Our estimates would be slightly smaller in this case. Fifth, the 1-compartment model assumes perfectly mixed conditions. However, rooms are often not perfectly mixed, and ventilation and room geometry will add spatiotemporal variability. The modeling provides an estimate, but exact concentrations will vary in function of the real circumstances. In multiroom situations, numerical flow simulations seem indicated to describe the microdroplet distribution.^48^ Sixth, although our results suggest that in certain situations, airborne transmission of COVID-19 may be possible, it is important to keep in mind that this was a modeling effort. While this route would provide a convenient explanation for several super-spreading events,^16,17,18,19^ and although the virus was found in airborne microdroplets in hospital situations,^20^ this route still needs to be validated in clinical settings and animal models.

## Conclusions

The results of our mathematical modeling suggest that the viral load in the air can reach critical concentrations in small and poorly ventilated rooms, especially when the individual is a super-spreader, defined as a person emitting large number of microdroplets containing a high viral load. However, people who are high emitters are not very common in the population and our findings suggest that only few people with very high viral load pose an infection risk in poorly ventilated closed environments. Nevertheless, strict respiratory protection is recommended whenever there is a chance to be in the same room with such an individual, particularly if they are coughing and especially when one is in the room for a prolonged period.
